# Exploratory analysis of interleukin‐38 in hospitalized COVID‐19 patients

**DOI:** 10.1002/iid3.712

**Published:** 2022-10-26

**Authors:** Dennis M. de Graaf, Lisa U. Teufel, Aline H. de Nooijer, Adriaan J. van Gammeren, Antonius A. M. Ermens, Ildikó O. Gaál, Tania O. Crișan, Frank L. van de Veerdonk, Mihai G. Netea, Charles A. Dinarello, Leo A. B. Joosten, Rob J. W. Arts, the Radboudumc Center for Infectious Diseases COVID‐19 Study Group

**Affiliations:** ^1^ Department of Internal Medicine, Radboud Institute of Molecular Life Sciences (RIMLS) and Radboudumc Center for Infectious Diseases Radboud University Medical Center Nijmegen The Netherlands; ^2^ Department of Medicine University of Colorado Aurora Colorado USA; ^3^ Department of Clinical Chemistry and Hematology Amphia Hospital Breda The Netherlands; ^4^ Department of Medical Genetics Iuliu Hatieganu University of Medicine and Pharmacy Cluj‐Napoca Romania; ^5^ Department of Immunology and Metabolism, Life and Medical Sciences Institute University of Bonn Bonn Germany

**Keywords:** COVID‐19, interleukin‐38, retrospective cohort study, SARS‐CoV‐2

## Abstract

**Introduction:**

A major contributor to coronavirus disease 2019 (COVID‐19) progression and severity is a dysregulated innate and adaptive immune response. Interleukin‐38 (IL−38) is an IL‐1 family member with broad anti‐inflammatory properties, but thus far little is known about its role in viral infections. Recent studies have shown inconsistent results, as one study finding an increase in circulating IL‐38 in COVID‐19 patients in comparison to healthy controls, whereas two other studies report no differences in IL‐38 concentrations.

**Methods:**

Here, we present an exploratory, retrospective cohort study of circulating IL‐38 concentrations in hospitalized COVID‐19 patients admitted to two Dutch hospitals (discovery *n* = 148 and validation *n* = 184) and age‐ and sex‐matched healthy subjects. Plasma IL‐38 concentrations were measured by enzyme‐linked immunosorbent assay, disease‐related proteins by proximity extension assay, and clinical data were retrieved from hospital records.

**Results:**

IL‐38 concentrations were stable during hospitalization and similar to those of healthy control subjects. IL‐38 was not associated with rates of intensive care unit admission or mortality. Only in men in the discovery cohort, IL‐38 concentrations were positively correlated with hospitalization duration. A positive correlation between IL‐38 and the inflammatory biomarker d‐dimer was observed in men of the validation cohort. In women of the validation cohort, IL‐38 concentrations correlated negatively with thrombocyte numbers. Furthermore, plasma IL‐38 concentrations in the validation cohort correlated positively with TNF, TNFRSF9, IL‐10Ra, neurotrophil 3, polymeric immunoglobulin receptor, CHL1, CD244, superoxide dismutase 2, and fatty acid binding protein 2, and negatively with SERPINA12 and cartilage oligomeric matrix protein.

**Conclusions:**

These data indicate that IL‐38 is not associated with disease outcomes in hospitalized COVID‐19 patients. However, moderate correlations between IL‐38 concentrations and biomarkers of disease were identified in one of two cohorts. While we demonstrate that IL‐38 concentrations are not indicative of COVID‐19 severity, its anti‐inflammatory effects may reduce COVID‐19 severity and should be experimentally investigated.

## Graphical abstract

A major contributor to coronavirus disease 2019 (COVID‐19) progression and severity is a dysregulated innate and adaptive immune response. Interleukin (IL‐38) is an IL‐1 family member with broad anti‐inflammatory properties, but thus far little is known about its role in viral infections. Here, we demonstrate that IL 38 concentrations are not indicative of COVID‐19 severity in hospitalized patients

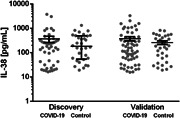

## 1 INTRODUCTION

Coronavirus disease 2019 (COVID‐19) is caused by infection with severe acute respiratory syndrome coronavirus 2 (SARS‐CoV‐2). Since the start of the outbreak in 2019, more than 273 million people have been infected as of December 2021, and over 5.3 million people have died worldwide.^1^ Clinical manifestation range from asymptomatic disease and mild symptoms to severe pneumonia, the development of acute lung injury mediated by exacerbated pathogenic inflammation, acute respiratory distress syndrome (ARDS), respiratory and multiorgan failure, and even death in a small but significant percentage of patients.^2^, ^3^

A dysregulated innate and adaptive immune response seems to be a major contributor to disease progression and severity.^2^, ^4^ For example, elevated concentrations of interleukin‐1β (IL‐1β), tumor necrosis factor (TNF), IL‐6, IL‐10, and IL‐1Ra were reported in COVID‐19 patients.^3^, ^5^ Notably, the concentration of the anti‐inflammatory IL‐1Ra and IL‐10 is positively correlated with disease severity.^6^ Depending on the timing and extent of cytokine production, the induction of anti‐inflammatory or the inhibition of proinflammatory cytokines during an infection can either limit the desirable inflammatory response aimed at clearing the pathogen or limit undesirable pathological inflammation that damages host tissues.^7^

In hospitalized COVID‐19 patients, therapeutic interventions entail, next to dexamethasone and monoclonal antibodies against SARS‐CoV‐2, such as casirivimab and imdevimab anti‐inflammatory drugs to control inappropriate immune responses.^8^ Anti‐inflammatory compounds currently used in the management of COVID‐19, for example, inhibit IL‐6 signalling (tocilizumab), interfere with JAK‐STAT signalling (baricitinib or tofacitinib), or inhibit the IL‐1 signalling pathway (anakinra).^8^

IL‐38 is an anti‐inflammatory member of the IL‐1 family that shares 41% and 43% sequence homology with the receptor antagonists IL‐1Ra and IL‐36Ra, respectively.^9^ IL‐38 is expressed in a variety of tissues including the basal epithelia of the skin, the heart, placenta, spleen, thymus, and activated B cells.^9^ Although IL‐38 lacks a caspase‐1 cleavage site, N‐terminal processing is required for biological activation.^10^, ^11^ As such, peripheral blood mononuclear cells (PBMCs) treated with full‐length IL‐38 produce more proinflammatory cytokines in response to lipopolysaccharide.^12^ Conversely, anti‐inflammatory responses are induced by truncated IL‐38 (20‐152) released from apoptotic macrophages resulting in attenuated JNK and activator protein‐1 (AP‐1) signalling and consequently reduced IL‐6 production by macrophages and T‐helper type 17 activation.^10^ Interestingly, exposure of PBMCs to low‐concentration, full‐length IL‐38 also limits T‐cell‐derived cytokine production upon *Candida albicans* stimulation, while higher concentrations moderately increase inflammatory responses.^12^ IL‐38 binds to the IL‐1R6 (*IL1RL2*),^12^ and the IL‐1R9 (*IL1RAPL1*)^10^ which is located on the X‐chromosome,^13^, ^14^ and in addition to the IL‐1R1 (*IL1R1*), although with lower affinity than IL‐1β or IL‐1Ra.^10^

With regard to its role in health and disease, aberrant IL‐38 concentrations have been observed in different diseases such as psoriasis, systemic lupus erythematosus, rheumatoid arthritis, Sjögren's syndrome, Crohn's disease, and myocardial infarction.^15^, ^16^, ^17^, ^18^, ^19^ IL‐38 has been studied in the context of pulmonary diseases; elevated IL‐38 concentrations have been measured in plasma of ARDS patients,^20^ as well as in the serum of patients with chronic obstructive pulmonary disease (COPD).^21^ In COPD, IL‐38 is also negatively correlated with C‐reactive protein (CRP), a marker for inflammation and fibrinogen, a biomarker of respiratory disease in COPD.^21^, ^22^ The therapeutic potential of IL‐38 to reduce inflammation‐mediated lung damage has been investigated in mouse models of lung disease or injury following cecal ligation and puncture,^20^ intranasal lipopolysaccharide (LPS),^20^ intraperitoneal poly(I:C) challenge,^23^ and bleomycin‐induced pulmonary fibrosis.^24^ Further, airway hyperreactivity in a model of allergic asthma was ameliorated by IL‐38 administration.^25^

Evidence of IL‐38's acute role in response to local or systemic infections, however, is scarce. In mice infected with coxsackievirus B3, neutralization of IL‐38 reduced survival and cardiac function and was associated with increased viral replication.^26^ In murine models of sepsis, IL‐38 administration decreased inflammatory cytokines and organ damage and augmented bacterial clearance.^27^ In humans, IL‐38 plasma concentrations were elevated in patients with sepsis, which negatively correlated to circulating proinflammatory cytokines and blood leukocyte counts.^27^ These data suggest that IL‐38 may be used in inflammatory conditions to alleviate symptoms. Interestingly, studies on human experimental endotoxemia demonstrated that bolus injection of LPS does not increase IL‐38 in an acute fashion.^28^ A study in patients with chronic hepatitis B, in which elevated serum IL‐38 concentrations reflected viral load and ongoing liver injury, supports that IL‐38 may rather play a role in the response to chronic infections.^29^

Gao et al.^23^ observed that circulating IL‐38 concentrations are increased in COVID‐19 patients compared to healthy controls. However, patients with severe disease have lower IL‐38 concentrations than patients with mild disease. Furthermore, IL‐38 in COVID‐19 patients was negatively correlated with hospitalization duration, CRP, and the inflammatory marker lactate dehydrogenase (LDH), but not with SARS‐CoV‐2 viral load in sputum or nasopharyngeal swab specimen.^23^ In contrast, Al‐Bassam et al.^30^ reported no differences in serum IL‐38 levels between patients and controls, and there was no difference between IL‐38 between moderate, severe, or critical patient status. These findings were corroborated by Kassianidis et al.,^31^ who additionally report that an increased IL‐38 concentration was detected in asymptomatic patients.

The aim of the current study is to investigate the role of circulating IL‐38 in hospitalized COVID‐19 patients and describe its relationship to clinical and inflammatory markers of disease. We hypothesize that reduced IL‐38 correlates with poor disease outcomes and is indicative of undesirable excessive inflammation that exacerbates COVID‐19 severity.

## 2 MATERIALS AND METHODS

### 2.1 Cohorts, sample collection, and processing

Samples from COVID‐19 patients were collected in two Dutch hospitals. Patients were included as described elsewhere.^4^ Briefly, patients admitted between March and April 2020 to the Amphia hospital in Breda (discovery cohort) and the Radboud University Medical Center in Nijmegen (validation cohort) in the Netherlands with clinically diagnosed SARS‐CoV‐2 infection were included in the study. The smaller group was assigned as the discovery cohort and the larger group as the validation cohort. Blood was collected in EDTA upon admission and after 72 h. If the hospital stay was longer, more time points were added. EDTA blood was centrifuged for 10 min at 2954 *g* at room temperature, and plasma was subsequently collected. Samples were stored at −80°C for cytokine analysis.

A control cohort consisting of 279 healthy individuals was used for comparisons, 204 of which had detectable IL‐38 values. Sample collection and processing were performed as described for the COVID‐19 patients. Controls were sex‐ and age‐matched (±4 years), resulting in the inclusion of 97 or 104 individuals for the discovery cohort and validation cohort, respectively. Blood was collected and processed as described earlier.

### 2.2 Data collection

For the discovery cohort, clinical data were collected as shown in Table 1. The date of hospital admission was assigned as Day 0 and total days of hospitalization were calculated based on the day of admission and the day of release or death.

For the validation cohort, laboratory results and clinical data were collected from the electronic patient files (EPIC; EPIC System) and recorded in electronic case report forms (Castor EDC). In addition to the patient characteristics, as shown in Table 1, information about required oxygen ([combination of]) none, nasal cannula, oxygen mask, venturi mask, non‐rebreathing mask, or invasive mechanical ventilation), the occurrence of ICU complications, baseline measurements of blood leukocyte counts assessed by XN‐45 hematology analyzer (Sysmex Corporation), procalcitonin, d‐dimer, CRP, creatinine, LDH, and ferritin were collected. The date of hospital admission was assigned as Day 0 and the first sample collected within 3 days of admission was considered a baseline measurement. Total days of hospitalization were calculated based on the day of admission and the day of release or death.

### 2.3 Proximity extension assay

Circulating protein abundance was assessed in plasma samples by proximity extension assay (OLINK Proteomics) as described previously.^4^ Ninety‐two inflammatory markers were analyzed in samples of the discovery cohort, and 276 inflammation‐, cardiometabolic‐, and cardiovascular disease (panel II)‐related markers were measured in samples of the validation cohort. Reads below a detection of 75% or with less than 20 valid reads were excluded from analyses, resulting in data on 69 and 232 proteins in total in the discovery cohort and validation cohort, respectively. Protein expression values are log 2‐transformed and represented as NPX (NeuroPhysiological signals in eXtensible Markup Language) data. Measurements for the validation cohort were performed in two batches and batch differences were corrected using bridging samples. Functional information about analyzed markers was obtained from Gene Ontology using AmiGO2,^32^ a tool for searching the Gene Ontology database.^33^, ^34^

### 2.4 Quantification of IL‐38 by ELISA

The human IL‐38/IL1F10 DuoSet ELISA (BioTechne) was used according to the manufacturer's instructions with minor adjustments regarding sample‐incubation time. Samples were incubated overnight at 4°C instead of 2 h at room temperature. Plasma samples were diluted 1:1 in phosphate‐buffered saline containing 1% bovine serum albumin (Sigma‐Aldrich, Germany). A standard curve of 7.8–2000 pg/ml yielded 15.6 pg/ml as the lower limit of quantification and 4000 pg/ml as the upper limit of quantification. Values below the detection limit were included in the analyses using the lower limit of quantification as the assigned value.

### 2.5 Statistical analyses

Statistical analyses were performed using R version 4.0.3 (RStudio), GraphPad Prism version 6 (GraphPad Software), and SPSS version 26.0 (IBM). Continuous data are represented as mean with standard deviation and nominal data are represented as count with percentages. IL‐38 was log‐transformed for all analyses and nominal data was assigned numerical coding, for example, 0 = deceased, 1 = recovered, and 2 = unknown.

Differences between the discovery and validation cohorts and the healthy controls were analysed using SPSS. Tests were chosen based on normality distribution and continuity of the data. Differences in age, BMI, and IL‐38 were assessed using one‐way analysis of variance, Welch *F*‐test was used for differences in hospitalization period, and the *χ* ^2^‐test for assessing the nominal variables ICU admission, sex, and mortality. The *χ* ^2^‐test was also used to determine differences in the ratio of IL‐38 above and below the limit of detection between the diseased and healthy cohorts. Data visualization and statistical analysis of differences in IL‐38 concentrations above detection limit as well as comparison of age and BMI between control and study cohorts were performed in GraphPad Prism using two‐tailed Mann–Whitney *U*‐test.

All remaining analyses, data wrangling, and visualization were performed using different R packages, for eample, dplyr, reshape, tidyr, psych, ggplot2, factoextra, ggstatsplot, ggpubr, grDevices, ggbiplot, ggrepel, and devtools. Correlations between patient characteristics and laboratory parameters, as well as between IL‐38 and clinical and laboratory parameters, markers of inflammation, cardio metabolism, and cardiovascular disease, were assessed by Spearman's rank test. *p* Values of correlations with more than five variables were corrected for multiple testing (false discovery rate [FDR] adjustment). All analyses except for the correlations with the marker panels were stratified by sex. Longitudinal evaluation of IL‐38 plasma concentrations was performed for samples above the detection limit using a Kruskal–Wallis test. Samples from 5 to 46 patients were included in the analyses for the validation cohort and discovery cohort, respectively. For comparison of IL‐38 concentrations based on days of hospitalization, total hospitalization period was binned into three groups based on the variable mean: “short hospitalization” (1–3 days), “medium hospitalization” (4–9 days), and “long hospitalization” (>9 days). Bins were stratified by sex and compared using Wilcoxon's test. Differentially expressed markers of inflammatory, cardiometabolic, and cardiovascular disease between patients who deceased and those who recovered were expressed as Volcano and PCA plots. *p* Values of the former were FDR adjusted.

## 3 RESULTS

### 3.1 General analyses of COVID‐19 cohorts

#### 3.1.1 Mortality is increased with age, but not with BMI

In total, plasma samples from 148 (discovery cohort) and 184 (validation cohort) patients with confirmed COVID‐19 were included in this study. Patient characteristics at hospital admission are provided in Table 1. Both cohorts were compared regarding age, sex, BMI, hospitalization period, intensive care unit (ICU) admission, IL‐38 plasma concentration, and mortality.

**Table 1:** Patient characteristics of discovery and validation cohort

	Discovery cohort			Validation cohort		
	Total	Male	Female	Total	Male	Female
Count	148	97/148 (66%)	51/148 (34%)	184	128/184 (70%)	56/184 (30%)
Age (years), M (±SD)	68 (±12)	69 (±12)	67 (±11)	63 (±13)	63 (±13)	62 (±13)
BMI, M (±SD)	28 (±5)	28 (±4)	29 (±6)	27 (±4)	27 (±4)	27 (±4)
Hospitalization period (days), M (±SD)	9 (±6)	10 (±7)	9 (±6)	6 (±4)	6 (±4)	6 (±3)
ICU admission	41/148 (28%)	30/97 (31%)	11/51 (22%)	77/184 (42%)	58/128 (45%)	19/56 (34%)
Mortality	44/148 (30%)	33/97 (34%)	11/51 (22%)	27/184 (15%)	18/128 (14%)	9/56 (16%)
IL‐38 (pg/ml), M (±SD)	125 (421)	126 (411)	124 (444)	137 (377)	121 (301)	173 (511)
Maximum	3735	3735	3035	3297	3297	1096
Minimum (detection limit)	15.6	15.6	15.6	15.6	15.6	15.6
Detectable	46/148 (32%)	30/97 (31%)	16/51 (31%)	61/184 (33%)	42/128 (33%)	19/56 (34%)
Undetectable	101/148 (68%)	67/97 (69%)	35/51 (69%)	123/184 (67%)	86/128 (67%)	37/56 (66%)

Abbreviations: BMI, body mass index; ICU, intensive care unit; IL, interleukin.

Sex was equally distributed between and within cohorts (discovery: 66% male; validation: 70% male, *χ* ^2^ (1) = 0.608, *p* = .435). Overall, more patients were admitted to the ICU in the validation cohort than in the discovery cohort (discovery: 28%; validation: 42%, *χ* ^2^ (1) = 7.164, *p* = .007), but admissions were equally distributed between and within cohorts when stratified by sex (discovery: 31% male and 22% female; validation: 45% male and 34% female, *χ* ^2^ (1) = 0.183, *p* = .669). Mortality was higher in the discovery than in the validation cohort (discovery: 30%; validation: 15%, *χ* ^2^ (1) = 11.059, *p* = .001); however, when stratified by sex, no differences were detected within and between cohorts (discovery: 34% male and 22% female; validation: 14% male and 16% female, *χ* ^2^ (1) = 2.046, *p* = .153). In the discovery cohort, both women and men were older (women: *F* (1, 105) = 5.67, *p* = .019; men: *F* (1, 223) = 11.68, *p* = .001) and had longer hospitalization periods (women: *F* (1, 77.42) = 11.75, *p* = .001; men: *F* (1, 142.24) = 17.71, *p* < .001) compared to the validation cohort. Further, women in the discovery cohort had higher BMI values (women: *F* (1, 100) = 4.87, *p* = .030) compared to women in the validation cohort. IL‐38 plasma concentrations were comparable between cohorts (*F* (1, 329) = .074, *p* = .784) and sexes (women: *F* (1, 104) = 0.284, *p* = .595; men: *F* (1, 223) = 0.009, *p* = .923).

**Figure 1:**
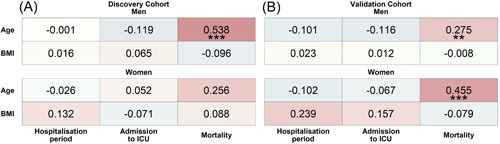
Association‐heatmap of age and BMI with hospitalization period, ICU admission, and mortality. Shown is a heatmap representation of Spearman's correlation coefficients and their significance for associations between patient characteristics of the discovery (A) and validation (B) cohort stratified by sex. Correlations are highlighted in color, with red representing a positive correlation and blue a negative correlation. Color intensity indicates Spearman's correlation coefficients (*r*). BMI, body mass index; ICU, intensive care unit. **p < .01; ***p < .001. Discovery_Women_ = 51, Discovery_Men_ = 97, Validation_Women_ = 56, and Validation_Men_ = 128.

Correlation analyses of both cohorts showed that while age was associated with increased mortality, BMI was not (Figure 1), confirming recently reported results.^35^ The association between age and mortality was significant in men but only trending toward significance in women of the discovery cohort and was significant for both sexes in the validation cohort.

Next, the associations between various inflammatory markers collected in the validation cohort and hospitalization period, ICU admission, and mortality were evaluated (Supporting Infomation: Table S1). Hospital admission positively correlated with d‐dimer in men and with d‐dimer and ferritin in women. Creatinine was associated negatively with hospitalization in women. ICU admission was positively associated with CRP, ferritin, d‐dimer, and procalcitonin in men and with LDH, d‐dimer, ferritin, and CRP in women. Again, creatinine was negatively correlated with ICU admission in women. Mortality was positively correlated with creatinine and procalcitonin in men and women, respectively.

### 3.2 Protein expression profiles are characteristic of COVID‐19 patients

Differential protein expression of biomarkers of inflammatory, cardiovascular, or cardiometabolic disease was compared between patients who deceased and recovered. We found various markers upregulated in deceased patients that have previously been associated with increased mortality and/or disease severity of COVID‐19. Proteins linked to severity are C–C chemokine motif chemokine 11 (CCL11), IL‐6, and IL‐10,^36^ lipoprotein lipase (LPL),^37^ vascular endothelial growth factor‐A,^38^ and vascular cell adhesion molecule‐1.^39^ Programmed death‐ligand 1,^40^ matrix metalloproteinase‐12,^41^ and cystatin C (CST3)^42^ have been linked to mortality, while adrenomedullin,^43^ placental growth factor,^38^ growth arrest‐specific 6,^44^ and fibroblast growth factor 21 (FGF21)^45^ are associated with both mortality and disease severity. Elevated renin (REN) has not been directly linked to mortality, but inhibition of the REN–angiotensin–aldosterone system decreases mortality of hypertensive COVID‐19 patients.^46^

Further, we identified several markers with reduced expression in deceased patients, five of which have been linked to COVID‐19: (1) CCL5 has been associated with reduced mortality^6^; (2) the anticoagulant protein C is increased in COVID‐19^47^; (3) FMS‐related receptor tyrosine kinase 3 ligand has been linked to disease severity but not to mortality^48^; (4) TNF‐related apoptosis‐inducing ligand TRAIL (*TNFSF10*) is elevated in women but not in men with severe COVID‐19,^49^ and (5) fetuin B is elevated in patients who recovered from COVID‐19 compared to those who deceased.^50^ Glyoxalase 1, LPL, TNFRSF10A and B, cystatin D (CST5), CCL19, FGF23, F11, cluster of differentiation 59, Fc gamma receptor IIa, carbonic anhydrase 3, regenerating family member 1α, oncostatin m receptor, plexin B2, C–X–C motif chemokine ligand 5, Delta/Notch‐like EGF repeat containing, interleukin‐6 family cytokine receptor subunit alpha (LIFR), CD40, CCL28, S100 calcium‐binding protein A12 (ENRAGE), CXCL1, CUB domain‐containing protein 1, and CCL11 are as of our knowledge described for the first time as differentially expressed between recovered and deceased COVID‐19 patients (Supporting Information: Figure S1).

### 3.3 Specific analyses of IL‐38 in hospitalized COVID‐19 patients

#### 3.3.1 Plasma IL‐38 concentrations of COVID‐19 patients are stable over time and akin to those of healthy subjects

To assess the stability of IL‐38 concentrations in COVID‐19 patients during hospitalization, plasma IL‐38 concentrations were measured at multiple timepoints in the discovery cohort up to 32 days after admission. IL‐38 remained stable during the entire hospitalization period (*χ* ^2^ (99) = 102.31, *p* = .38). In the validation cohort, samples from three timepoints between 1 and 6 days upon admission were measured, and IL‐38 stability over time was confirmed (*χ* ^2^ (8) = 9, *p* = .342).

To evaluate whether IL‐38 is altered in COVID‐19 patients, IL‐38 plasma concentrations in both cohorts were compared to a age‐ and sex‐matched healthy control cohort (Table 2). Demographically, age (*U* = 6092, *n*_discovery_ = 148, *n*_control_ = 97, *p* = .045 two‐tailed) but not body mass index (BMI) (*U* = 5624, *n*_discovery_ = 139, *n*_control_ = 95, *p* = .053 two‐tailed) differed between the discovery and control cohorts. In the validation cohort, BMI (*U* = 7455, *n*_validation_ = 178, *n*_control_ = 103, *p* = .009 two‐tailed) but not age (*U* = 8890, *n*_validation_ = 184, *n*_control_ = 104, *p* = .39 two‐tailed) differed from the control cohort. Downstream stratification by sex revealed that women of the discovery cohort and of the validation cohort were statistically comparable regarding age (*U* = 1269, *n*_discovery_ = 51, *n*_control_ = 50, *p* = .966; *U* = 1412, *n*_validation_ = 56, *n*_control_ = 52, *p* = .789 two‐tailed) and BMI (*U* = 1099, *n*_discovery_ = 49, *n*_control_ = 49, *p* = .469; *U* = 1143, *n*_validation_ = 53, *n*_control_ = 52, *p* = .132 two‐tailed) with the corresponding healthy controls. Men in the discovery cohort were older than controls (discovery cohort 69 ± 12 vs. control cohort 64 ± 9 years; *U* = 1663, *n*_discovery_ = 97, *n*_control_ = 47, *p* = .008 two‐tailed) but did not differ in BMI (*U* = 1716, *n*_discovery_ = 90, *n*_control_ = 46, *p* = .102 two‐tailed). Men in the validation cohort had lower BMI (validation cohort 27 ± 4 vs. control cohort 28 ± 4; *U* = 2513, *n*_validation_ = 125, *n*_control_ = 51, *p* = .027 two‐tailed) but did not differ in age (*U* = 2936, *n*_validation_ = 128, *n*_control_ = 52, *p* = .216 two‐tailed) compared to their respective matched healthy controls (Table 2 and Supporting Information: Figure S3).

**Table 2:** Comparison of discovery and validation cohorts with corresponding healthy, age‐ and sex‐matched control cohorts

	Healthy control cohort for the discovery cohort			Healthy control cohort for the validation cohort		
	Total	Male	Female	Total	Male	Female
Count	97	47/97 (48%)	50/97 (52%)	104	52/104 (50%)	52/105 (50%)
Age (years), M (± SD)	66 (±10)	64 (±9)	68 (±11)	62 (±10)	61 (±11)	63 (±10)
BMI, M (± SD)	29 (±4)	29 (±4)	29 (±4)	28 (±4)	28 (±4)	28 (±5)

Abbreviation: BMI, body mass index.

**Figure 2:**
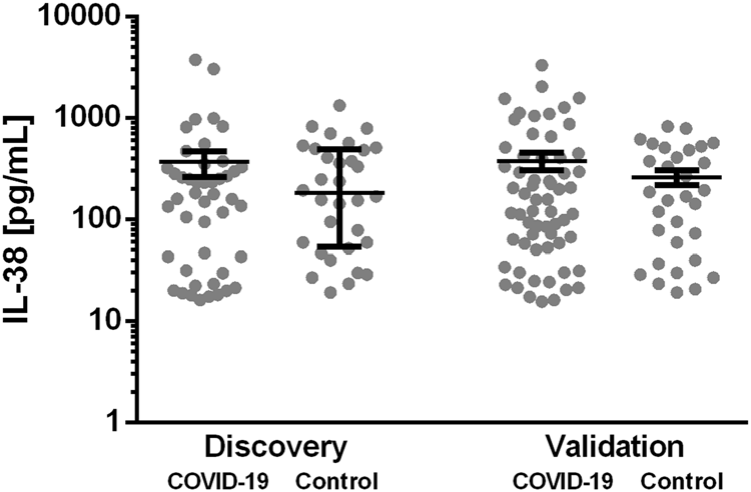
IL‐38 plasma concentration in COVID‐19 cohorts compared to sex‐ and age‐matched healthy control subjects. Circulating IL‐38 plasma concentrations of COVID‐19 patients were compared to healthy controls by Mann–Whitney *U*‐test after exclusion of individuals with IL‐38 concentrations below the detection limit. Data are displayed as median ± IQR. COVID‐19, coronavirus disease 2019; IL, interleukin. Discovery_COVID‐19_ *n* = 46, Discovery_Control_ *n* = 31, Validation_COVID‐19_ *n* = 61, and Validation_Control_ *n* = 32.

Stratified by sex, the ratio of detectable to undetectable IL‐38 values was equal between cohorts. Among subjects with detectable IL‐38 plasma concentrations, there was no difference between the COVID‐19 patients and matched healthy subjects (*U* = 640, *n*_discovery_ = 46, *n*_Control_ = 31, *p* = .452 two‐tailed; *U* = 2537, *n*_validation_ = 61, *n*_control_ = 32, *p* = .755 two‐tailed) (Figure 2).

Exploring the relation of IL‐38 with patient characteristics, a negative correlation between IL‐38 and BMI was identified in the validation cohort in a sex‐dependent manner (men: Spearman's *r* = −0.24, *p* = .007; women: Spearman's *r* = −0.14, *p* = .75). No correlation between IL‐38 and age was found. IL‐38 was also not associated with age in the matched healthy controls (Spearman's *r*_discovery_ = 0, *n* = 97, *p* = .98; Spearman's *r*_validation_ = .02, *n* = 104, *p* = .87) nor in the complete healthy control cohort (Spearman's *r* = −0.1, *n* = 204, *p* = .15).

### 3.4 IL‐38 plasma concentrations are not associated with the clinical outcome of COVID‐19

**Figure 3:**
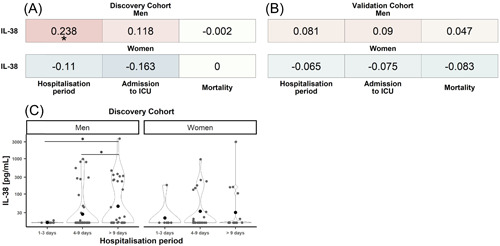
Association‐heatmap between IL‐38 and hospitalization period, ICU admission, and mortality and relation of IL‐38 to hospitalization. Shown is a heatmap representation of Spearman's correlation coefficients and their significance for associations between patient characteristics of the discovery (A) and validation (B) cohort stratified by sex Correlations are highlighted in color, with red representing a positive correlation and blue a negative correlation. Color intensity indicates Spearman's correlation coefficients (*r*). *p < .05. Discovery_Women_ = 51, discovery_Men_ = 97, validation_Women_ = 56, and validation_Men_ = 128. (C) A comparison of IL‐38 concentrations between artificial bins of the hospitalization period based on the variable mean (“short hospitalization” [1–3 days], “medium hospitalization” [4–9 days], and “long hospitalization” [>9 days]) stratified by sex using Wilcoxon's test. ICU, intensive care unit; IL, interleukin. *p < .05. 1–3 days_Women_ = 8, 1–3 days_Men_ = 9, 4–9 days_Women_ = 24, 4–9 days_Men_ = 54, >9 days_Women_ = 19, and >9 days_Men_ = 34.

Correlation analyses of IL‐38 with mortality and clinical markers of disease severity showed no relationship of the cytokine with mortality, ICU admission, or hospitalization period. No relationship between IL‐38 with ICU admission nor with mortality was found after stratification by sex (Figure 3). However, a small yet significant positive correlation of IL‐38 with the duration of hospitalization in men of the discovery cohort was identified (Figure 3A), which was however not replicated in the validation cohort (Figure 3B).

In‐depth analysis of the hospitalization period revealed that IL‐38 concentrations in men of the discovery cohort did not differ between “short hospitalization period” (1–3 days) and “medium hospitalization” (4–9 days). However, differences between “short hospitalization” and “long hospitalization” (*p* = .038), and “medium hospitalization” and “long hospitalization” (*p* = .036) were detected (Figure 3C). This indicates that the correlation is driven by male patients who stayed longer than 9 days in the hospital. No differences were found in the validation cohort nor for women in the discovery cohort.

### 3.5 IL‐38 plasma concentrations correlate with d‐dimer in men with thrombocyte counts in women

**Figure 4:**
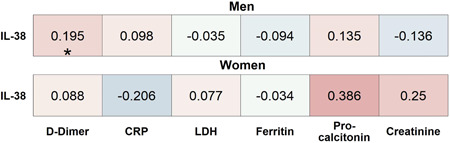
Association‐heatmap between IL‐38 and clinically measured biomarkers of disease in the validation cohort. Shown is a heatmap representation of Spearman's correlation coefficients and their significance for associations between patient characteristics stratified by sex. Correlations are highlighted in color, with red representing a positive correlation and blue a negative correlation. Color intensity indicates Spearman's correlation coefficients (*r*), and *p* values are FDR‐adjusted. CRP, C‐reactive protein; FDR, false discovery rate; IL, interleukin; LDH, lactate dehydrogenase. *p < .05. Validation_Women_ *n* = 56 and Validation_Men_ *n* = 128.

Analyses of the relationship between IL‐38 to various inflammatory markers and clinical parameters of the validation cohort revealed a positive correlation between IL‐38 plasma concentrations and d‐dimer in men, but not in women, and no relationship with CRP, LDH, ferritin, procalcitonin, and creatinine (Figure 4). Notably, IL‐38 correlated negatively with thrombocytes in women but not in men (Supporting Information: Figure S2). None of the other clinical parameters that were analyzed including blood leukocyte subset numbers, ICU complications, and oxygen supplementation was significantly correlated to IL‐38 (Supporting Information: Figure S2).

### 3.6 IL‐38 plasma concentrations are linked to circulating biomarkers

Next, the relationship of IL‐38 in COVID‐19 with all measured biomarkers was assessed in an exploratory correlation analysis in that identified 11 circulating proteins linked to IL‐38 (Table 3). Interestingly, superoxide dismutase 2 (SOD2), one of the markers with reduced expression in deceased patients compared to recovered patients, was also positively correlated to IL‐38. After correction for multiple testing, TNFRSF9, polymeric immunoglobulin receptor (PIgR), and neurotrophil 3 (NT‐3) remained significantly associated with circulating IL‐38.

**Table 3:** Correlation of circulating IL‐38 with biomarkers of disease

Protein ID (full protein name)	Function	Spearman's *r*	Unadj. *p* value	Adj. *p* value
NT‐3 (neurotrophin‐3)	Nervous system development (GO:0007399) Positive chemotaxis (GO:0050918)	0.336	.002	.008
TNFRSF9 (TNF receptor superfamily member 9)	Negative regulation of cell population proliferation (GO:0008285) Tumor necrosis factor‐mediated signalling pathway (GO:0033209)	0.270	.008	.030
PIgR (polymeric immunoglobulin receptor)	Mediated by polymeric immunoglobulin receptor (GO:0002415) Fc receptor signalling pathway (GO:0038093)	0.297	.009	.047
COMP (cartilage oligomeric matrix protein)	Extracellular matrix organization (GO:0030198) Positive regulation of chondrocyte proliferation (GO:1902732)	−0.240	.018	.053
CHL1 (neural cell adhesion molecule L1‐like protein)	Cell adhesion (GO:0007155) Axon guidance (GO:0007411)	0.229	.024	.068
TNF (tumor necrosis factor)	Positive regulation of cytokine production involved in inflammatory response (GO:1900017) Positive regulation of fever generation (GO:0031622)	0.231	.024	.072
SERPINA12 (serpin family A member 12)	Negative regulation of endopeptidase activity (GO:0010951)	−0.261	.023	.088
CD244 (CD244)	Adaptive immune response (GO:0002250) Leukocyte migration (GO:0050900)	0.22	.032	.088
IL10RA (interleukin 10 receptor subunit alpha)	Interleukin‐10 binding (GO:0019969)	0.243	.035	.095
	Negative regulation of autophagy (GO:0010507)			
SOD2 (superoxide dismutase 2)	Superoxide dismutase activity (GO:0004784) Negative regulation of oxidative stress‐induced intrinsic apoptotic signalling pathway (GO:1902176)	0.252	.028	.102
FABP2 (fatty acid‐binding protein 2)	Triglyceride catabolic process (GO:0019433) Intestinal lipid absorption (GO:0098856)	0.252	.028	.103

Abbreviation: IL, interleukin.

It is important to note that the correlations for TNFRSF9, TNF, and CD244 were only identified in the validation cohort but not in the discovery cohort. Measurements for IL‐10RA in the discovery cohort were excluded due to low quality. The markers of the cardiometabolic and cardiovascular panel were only available for the validation cohort.

## 4 DISCUSSION

It has been postulated that individual immune characteristics influence the development of a maladaptive systemic inflammatory response in COVID‐19.^51^, ^52^ As an anti‐inflammatory cytokine, we hypothesized that IL‐38 is indicative of reduced disease severity and mortality in COVID‐19 by limiting excessive inflammation caused by the infection. On the other hand, IL‐38 may dampen immune cell activity required for, for example, pathogen clearance in the early stages upon infection. Here, we provide an exploratory analysis of IL‐38 plasma concentrations in two cohorts of hospitalized COVID‐19 patients. Our study provides four major findings. First, the IL‐38 plasma concentration is not altered in COVID‐19 patients compared to age‐ and sex‐matched, healthy controls and remains stable over the period of hospitalization. Second, IL‐38 is not associated with disease severity and mortality, but we observed a positive association between IL‐38 and a prolonged hospitalization period in men of the discovery cohort. However, this was not confirmed in the larger validation cohort and may be an accidental finding. Third, the thromboinflammatory marker d‐dimer correlated positively with IL‐38 in men of the validation cohort. Fourth, IL‐38 correlated negatively with circulating thrombocyte counts in women of the validation cohort. Further, there was no association between IL‐38 and complications in the ICU, leukocyte subsets, or the requirement of oxygen treatment. Together, our data indicate that our hypothesis of reduced IL‐38 being indicative of increased COVID‐19 severity was not confirmed. However, it is noteworthy that the validation cohort consisted of younger patients with shorter hospitalization periods in comparison to the discovery cohort.

The stability of IL‐38 in COVID‐19 patients and the comparable IL‐38 concentrations between diseased and healthy subjects are in line with a study on IL‐38 in COVID‐19 patients by Al‐Bassam et al.,^30^ who have recently reported no differences in serum IL‐38 concentrations between healthy subjects and patients, nor differences between patients with moderate, severe, or critical disease. Our data are also in agreement with a study on healthy subjects that reported stable IL‐38 concentrations over the course of 1 year and unaffected IL‐38 concentrations in experimental human endotoxemia,^28^ and would imply that IL‐38 concentrations are unaffected by COVID‐19. Further more, Kassianidis et al.^31^ reported that patients with moderate or severe COVID‐19 have comparable serum IL‐38 concentrations as healthy control subjects while observing that asymptomatic COVID‐19 patients had increased IL‐38 concentrations.

In contrast, the report of Gao et al.^23^ indicated that circulating IL‐38 is increased in COVID‐19 patients compared to healthy controls, but reduced in severe patients compared to mild patients. Further, our results concerning the effect of IL‐38 on hospitalization period do not confirm the recent data from Gao et al.,^23^ who observed a negative correlation between serum IL‐38 concentrations of COVID‐19 patients with disease severity and inflammation, as well as with serum CRP, LDH, and hospitalization duration. In contrast, we observed a positive correlation between IL‐38 and the hospitalization period in men in the discovery cohort. The correlation between IL‐38 and hospitalization observed in the validation cohort was also positive, but did not reach statistical significance.

We consider several explanations for the discrepancies between our data and those reported by Gao et al. First, the expression of IL‐38 over the course of an infection may change, and thus the timing of the blood draw is relevant. However, in hospitalized COVID‐19 patients, we observe that IL‐38 concentrations were indeed stable. Furthermore, different ethnicities (Chinese vs. Dutch patients) may contribute to the opposing observations between our and Gao's study. A more likely explanation for the discrepancy between our studies is the difference in age. Gao et al. studied 85 relatively young patients with a mean age of 38 ± 17 (mean ± SD) years, while patients studied in our discovery cohort were 68 ± 12 years old, and patients in the validation cohort were 63 ± 13 years of age. We and others describe a positive association between age and mortality in COVID‐19 patients.^35^ The lower age in the validation cohort and potentially consequent reduced overall mortality (discovery: 30%; validation: 15%) and hospitalization period (discovery: 9 (±6) days; validation: 6 (±4)) may have confounded the effect of IL‐38 on hospitalization duration and severity. Moreover, patients studied by Gao et al. had overall less severe COVID‐19 than the two cohorts presented here, based on the reduced need for oxygen supplementation (discovery cohort: unknown; validation cohort: 93.5%; Gao et al.: 5.8%) as well as lower mortality (discovery cohort: 30%; validation cohort: 15%; Gao et al.: 0%).

Coronaviruses are recognized by Toll‐like receptor‐3 (TLR3), TLR2, and TLR7, which activate the innate immune system^53^ and induce antiviral cytotoxic immune activity.^54^ Gao et al. used poly(I:C) as a stimulus for TLR3 to mimic coronavirus‐induced lung inflammation in mice and observed that treatment with recombinant IL‐38 reduced the influx of inflammatory leukocytes, and inhibited induction of proinflammatory cytokines and chemokines.^23^ It is thus possible that elevated IL‐38 plasma concentrations reduce inflammatory responses by reducing TLR3 signalling, blocking the consequent antiviral immune response in the initial stages of the disease. An animal model for COVID‐19 that employs IL‐38 deficient mice compared to WT mice would clarify the role of endogenous IL‐38 in the disease process, and its therapeutic benefits can be explored using recombinant IL‐38. Another study design that would further clarify the role of IL‐38 in COVID‐19 or other viral infections is one that measures IL‐38 well before disease onset, in comparison to our study in which IL‐38 was measured shortly after hospitalization.

A potential confounder for the detection of IL‐38 may be due to differences between plasma and serum. However, we recently demonstrated that the blood collection method does not influence the IL‐38 concentrations detected by enzyme‐linked immunosorbent assay (ELISA).^55^

We further observed a positive correlation between d‐dimer and IL‐38 in men of the validation cohort. Elevated d‐dimer is a prognostic marker for severe COVID‐19,^56^ as well as for thrombotic complications, and is associated with increased proinflammatory cytokine levels such as IL‐6.^57^, ^58^ The correlation between IL‐38 and d‐dimer implicates that a subject with high IL‐38 might be prone to develop severe disease and COVID‐19‐associated complications, resulting in elevated, circulating d‐dimer. However, considering the observational nature of this study, high IL‐38 within the patient group can also reflect an ongoing attempt of the immune system to limit d‐dimer or the breakdown of an established clot.

An additional observation that may link IL‐38 to clotting is the negative association between IL‐38 and thrombocyte counts in women of the validation cohort. In COVID‐19, thrombosis is a frequently occurring symptom.^59^, ^60^ Yet, merely based on our observations we cannot conclude that IL‐38 reduces thrombosis risk in SARS‐CoV‐2 infected women. However, our data imply for the first time a role for IL‐38 in platelet biology. Whether a general inhibitory effect of IL‐38 on platelets and clotting exists remains to be experimentally assessed. As IL‐1β is associated with high platelet numbers,^61^ we consider plausible that IL‐38 inhibits the autocrine loop between IL‐1β and platelets.

Analyses of inflammation‐, cardiometabolic‐, and cardiovascular disease‐related markers yielded positive correlations between IL‐38 and several COVID‐19‐associated circulating proteins. CD244, which maintains an exhausted phenotype in natural killer (NK) cells and T cells, is upregulated in COVID‐19 patients, indicating that cytotoxic effector cells are dysfunctional in COVID‐19.^62^ IL‐38 may contribute to effector‐cell exhaustion by elevating CD244 expression. Whether NK‐ and T‐cell exhaustion contribute to disease severity remains to be investigated. Another predictor of COVID‐19 severity is elevated TNF,^2^ which is positively correlated to IL‐38. This could suggest that TNF induces IL‐38 to limit inflammation. In addition, IL‐10 is a biomarker for COVID‐19 severity.^6^, ^63^ The positive correlation between IL‐10RA and IL‐38 may indicate that IL‐38 induces the expression of this anti‐inflammatory receptor. The elevated expression of IL‐10 and its receptor IL‐10RA may be a mechanism to limit the cytokine storm. In inflammatory conditions, it is common that proinflammatory cytokines induce their antagonist^64^, ^65^ as observed for IL‐1Ra and IL‐10 in COVID‐19.^6^ Whether IL‐38 remains stable throughout the course of the disease or is induced upon infection is an open question. Moreover, the mediators that induce IL‐38 expression are unknown. Lastly, it remains to be investigated whether these markers also correlate with IL‐38 in healthy individuals.

TNFRSF9, SOD2, PIgR, cartilage oligomeric matrix protein, SERPINA12, fatty acid binding protein 2, neural cell adhesion molecule L1 like (CHL1), and NT‐3 were found to be associated with IL‐38, yet have not been described before in the context of COVID‐19 or IL‐38 biology. Our analysis showed that SOD2 expression was reduced in nonsurvivors compared to survivors, which could relate to elevated tissue damage through oxidative stress. The correlation of IL‐38 with CHL1 and NT‐3, both of which are involved in neuroplasticity and memory, could indicate a role for IL‐38 in the brain. However, as neither CHL1 nor NT‐3 have been implied with COVID‐19, for example, in the context of long‐lasting, cognitive COVID‐19 symptoms such as memory loss and “brain fog,”^66^ their specific role in relation to long‐term effects of COVID‐19 and IL‐38 can only be determined with further research.

Finally, various studies have reported a higher COVID‐19 incidence and mortality in men.^67^ Although the underlying causes are not yet fully understood, accumulating evidence points toward sex‐associated differences in SARS‐CoV‐2‐induced immune responses.^49^ Our results suggest that IL‐38 may be a contributing factor to sex differences in disease outcome of COVID‐19, as the positive associations of IL‐38 with d‐dimer and hospitalization were only found in men, while the negative association of IL‐38 with thrombocytes was only found in women. Our analyses further show that IL‐38 concentrations are not elevated in hospitalized COVID‐19 patients when compared to healthy individuals and that concentrations remain stable during hospitalization. Instead, based on the observed positive associations between IL‐38 and COVID‐19‐related biomarkers as well as prolonged hospitalization, we consider it plausible that individuals with high IL‐38 may have suppressed immune activity already before contracting SARS‐CoV‐2. Although it has been reported in chronic hepatitis B that increased IL‐38 is associated with enhanced viral clearance upon antiviral therapy,^29^ high baseline IL‐38 concentrations may promote a delayed immune stimulation in the first phase of COVID‐19, thereby dampening the initial antiviral immunity and promoting over‐induction of proinflammatory mediators and severe symptoms in the late stage of the infection. Unfortunately, we did not have access to data on viral load, and this hypothesis merits further investigation. On the other hand, in individuals with severe inflammatory conditions, IL‐38 may be used as a treatment to improve symptoms and the outcome of the patients. The role of IL‐38 in viral and infectious diseases warrants further follow‐up.

## AUTHOR CONTRIBUTIONS

*Conceptualization (ideas, research goals, and aims)*: Dennis M. de Graaf and Charles A. Dinarello. *Methodology (development or design of methodology; creation of models)*: Dennis M. de Graaf, Lisa U. Teufel, and Leo A. B. Joosten. *Investigation (conducting research, specifically performing the experiments, or data collection)*: Dennis M. de Graaf, Lisa U. Teufel, Aline H. de Nooijer, Antonius A. M. Ermens, Adriaan J. van Gammeren, Ildikó O. Gaál, and Tania O. Crișan. *Validation (verification of the overall reproducibility of results)*: Dennis M. de Graaf, Lisa U. Teufel, and Aline H. de Nooijer. *Formal analysis (application of statistical, mathematical, and computational techniques to analyze study data)*: Lisa U. Teufel. *Visualization (preparation, creation of visualization, and data presentation)*: Dennis M. de Graaf and Lisa U. Teufel. *Data curation/management (activities to annotate, scrub data, and maintain research data)*: Lisa U. Teufel. *Writing – original draft (preparation, creation, and presentation, specifically writing the initial draft)*: Dennis M. de Graaf and Lisa U. Teufel. *Writing – review and editing (critical review, commentary, or revision)*: Dennis M. de Graaf, Lisa U. Teufel, Charles A. Dinarello, Leo A. B. Joosten, Mihai G. Netea, and Rob J. W. Arts. *Project administration (management and coordination responsibility for the research activity)*: Dennis M. de Graaf, Lisa U. Teufel, and Rob J. W. Arts. *Supervision (oversight and leadership responsibility for the research activity planning and execution)*: Rob J. W. Arts, Leo A. B. Joosten, Mihai G. Netea, Charles A. Dinarello. *Resources (provision of study materials, reagents, materials, patients, laboratory samples, instrumentation, computing resources, or other analysis tools)*: Antonius A. M. Ermens, Adriaan J. van Gammeren, Ildikó O. Gaál, Tania O. Crișan, Frank L. van de Veerdonk, Charles A. Dinarello, Leo A. B. Joosten, and Mihai G. Netea. *Funding acquisition (acquisition of the financial support for the project leading to this publication)*: Charles A. Dinarello, Leo A. B. Joosten, Mihai G. Netea, and Rob J. W. Art.

## CONFLICT OF INTEREST

The authors declare no conflict of interest.

## ETHICS STATEMENT

The study was conducted in accordance with the Declaration of Helsinki. The study was approved by the local ethics committees (validation cohort from Nijmegen, the Netherlands: CMO 2020 6344 and CMO 2016 2963, and healthy control subjects from Cluj‐Napoca, Romania: 425/24.11.2016). Samples from the discovery cohort (Breda, the Netherlands) concerned secondary use of already collected blood samples, therefore no ethical approval was necessary. Before enrollment, written informed consent was obtained from participants from the discovery and control cohorts. Patients from the validation cohort or their legal representatives were informed about the study details and could decline to participate.^4^

## RADBOUDUMC CENTER FOR INFECTIOUS DISEASES COVID‐19 STUDY GROUP

Anneke Hijmans, Bram van Cranenbroek, Chantal Bleeker‐Rovers, Cor Jacobs, Emma Kooistra, Esther Fasse, Esther van Rijssen, Esther Taks, Fieke Weren, Gerine Nijman, Hans Koenen, Heidi Lemmers, Heiman Wertheim, Helga Dijkstra, Hetty van der Eng, Hidde Heesakkers, Ilse Kouijzer, Inge Grondman, Irma Joosten, Jaap ten Oever, Jacobien Hoogerwerf, Janette Rahamat‐Langendoen, Jelle Gerretsen, Jeroen Schouten, Joost Hopman, Josephine van de Maat, Kiki Schraa, Leonie Buijsse, Liesbeth van Emst, Liz Fransman, Manon Kolkman, Margreet Klop‐Riehl, Martin Jaeger, Nico Janssen, Nicole Waalders, Niklas Bruse, Noortje Rovers, Pleun Hemelaar, Priya Debisarun, Quirijn de Mast, Reinout van Crevel, Remi Beunders, Ruben Smeets, Simone Moorlag, Sjef van der Velde, Tim Frenzel, Tirsa van Schaik, Trees Jansen, and Wout Claassen. All these authors are affiliated with the Radboudumc Center for Infectious Diseases.

## Supporting information

Supplementary information.

Supplementary information.

Supplementary information.

Supplementary information.

## Data Availability

The data that support the findings of this study are available from the corresponding author upon reasonable request.
