# Supplementary figures and images for: Exploratory analysis of interleukin‐38 in hospitalized COVID‐19 patients
Source: Immun Inflamm Dis. 2022 Oct 26;10(11):e712. doi: 10.1002/iid3.712 (PMC9601778; doi:10.1002/iid3.712)

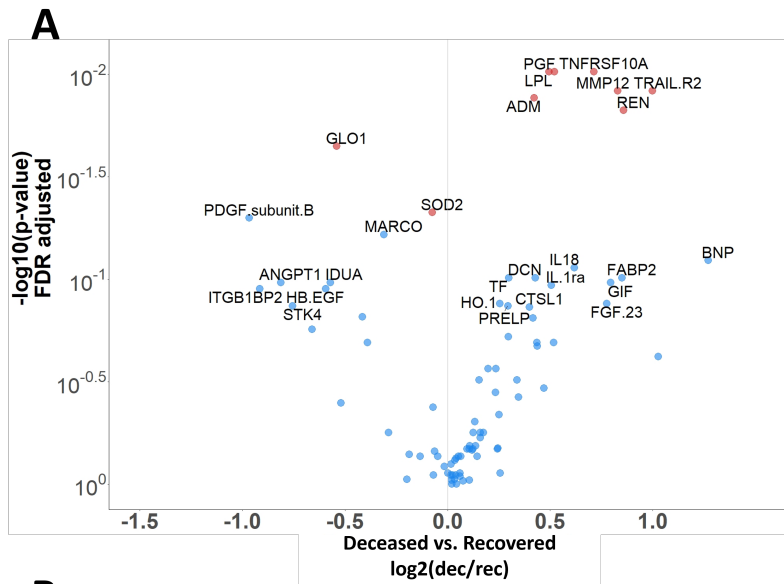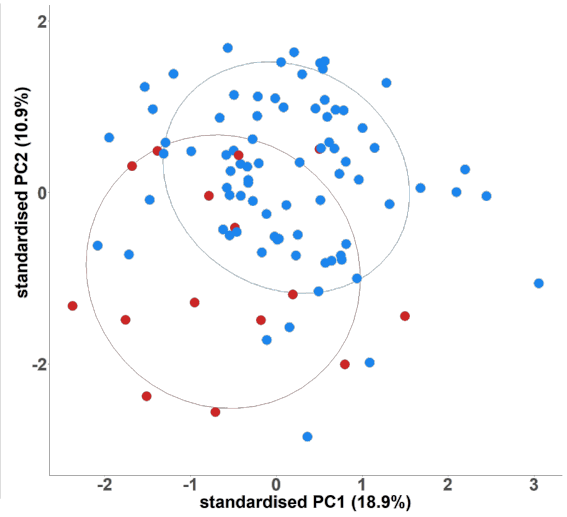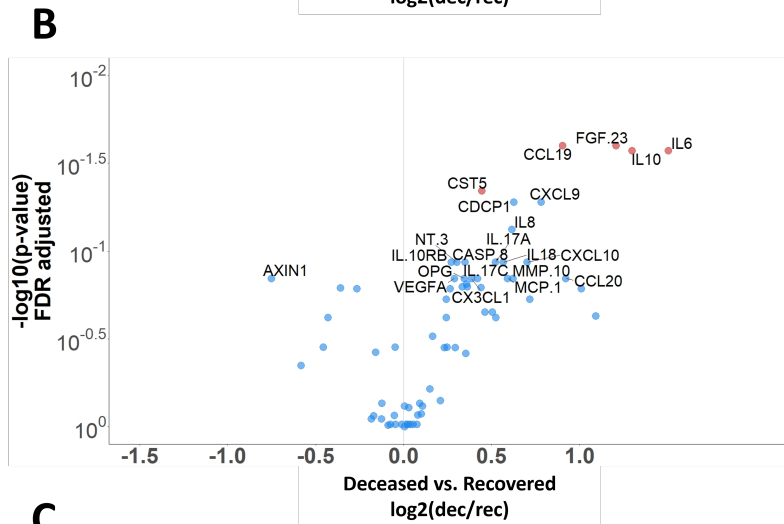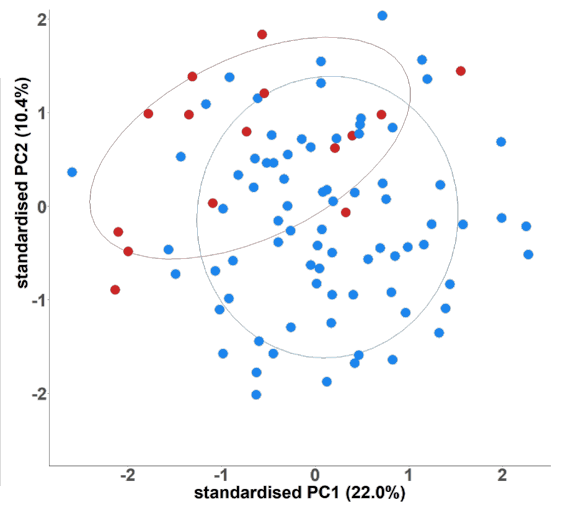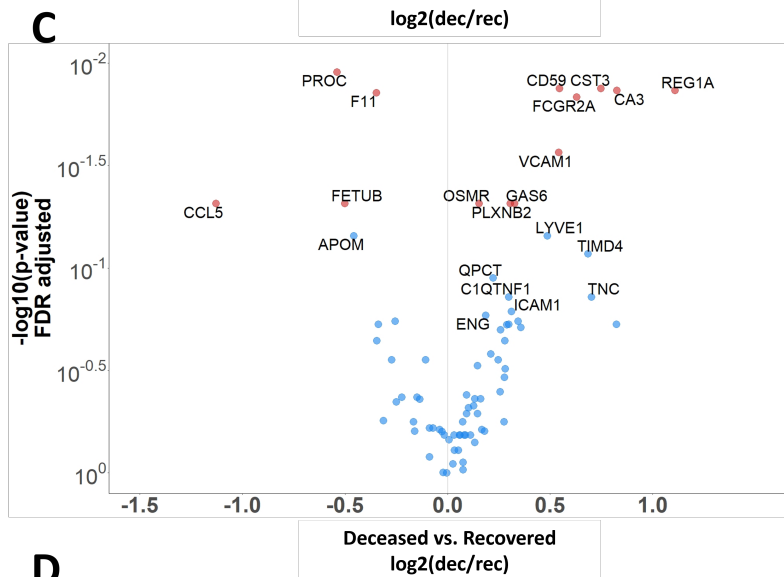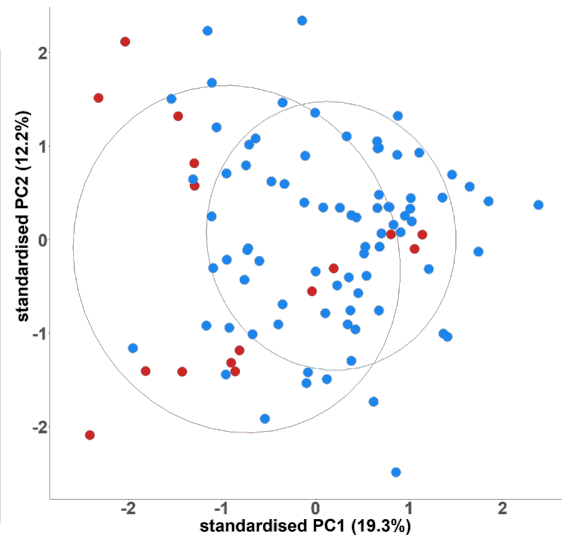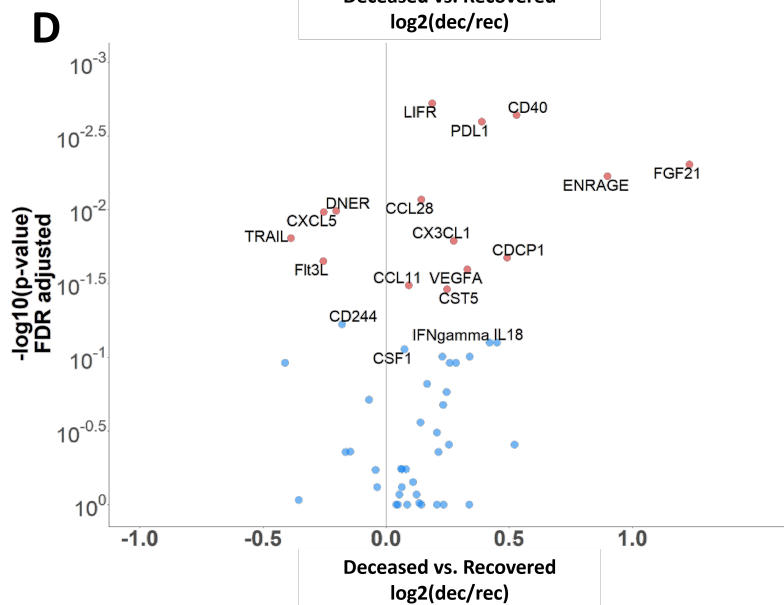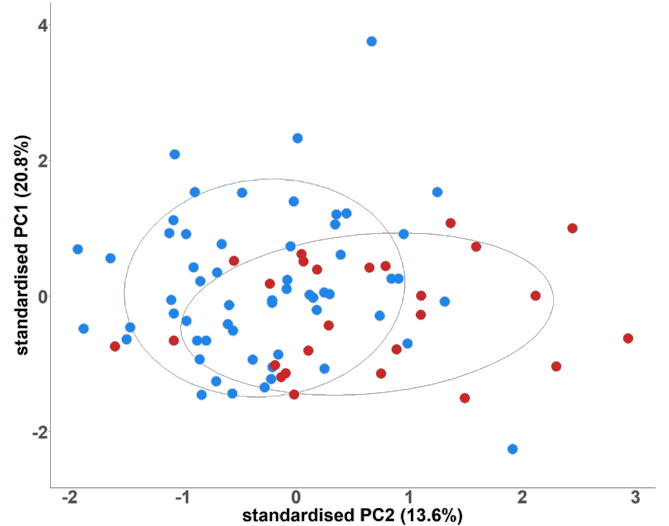

Supplement: Supplementary file 1 — Supplementary information. [file IID3-10-0-s002.pdf]

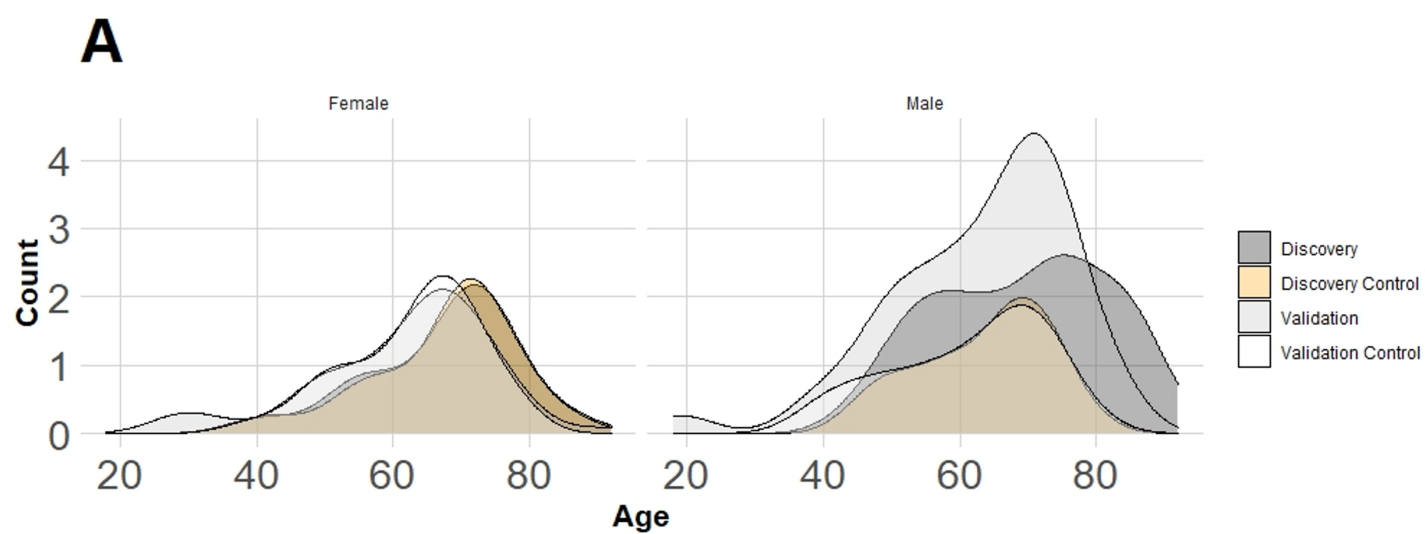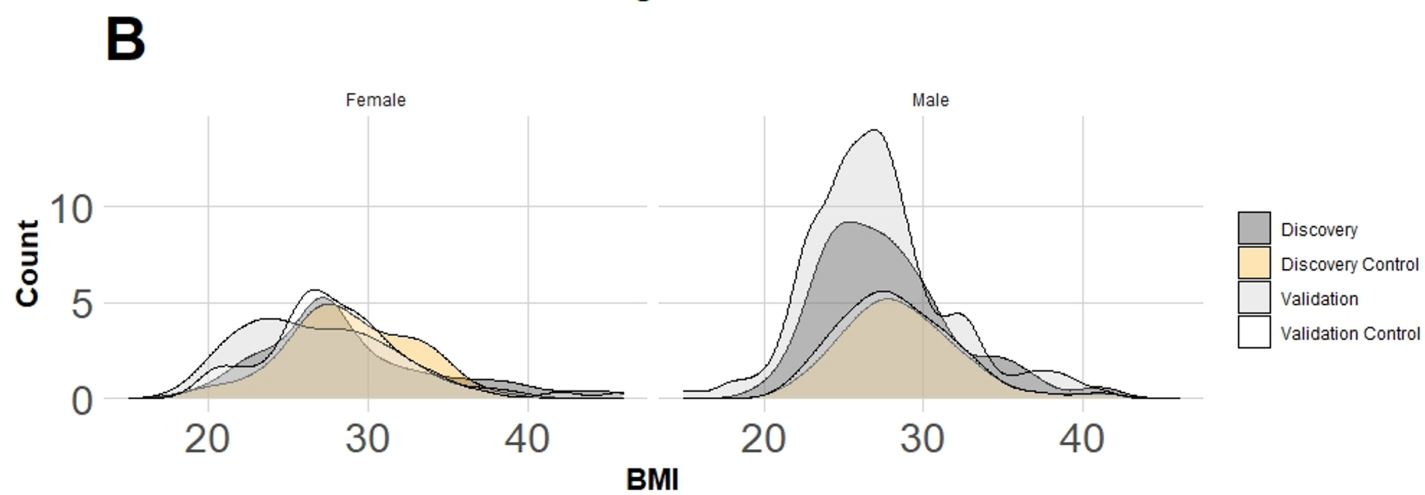

Supplement: Supplementary file 3 — Supplementary information. [file IID3-10-0-s004.pdf]
