# Supplementary material for: Exploratory analysis of interleukin‐38 in hospitalized COVID‐19 patients
Source: Immun Inflamm Dis. 2022 Oct 26;10(11):e712. doi: 10.1002/iid3.712 (PMC9601778; doi:10.1002/iid3.712)
Supplement: Supplementary file 2 — Supplementary information. [file IID3-10-0-s001.pdf]

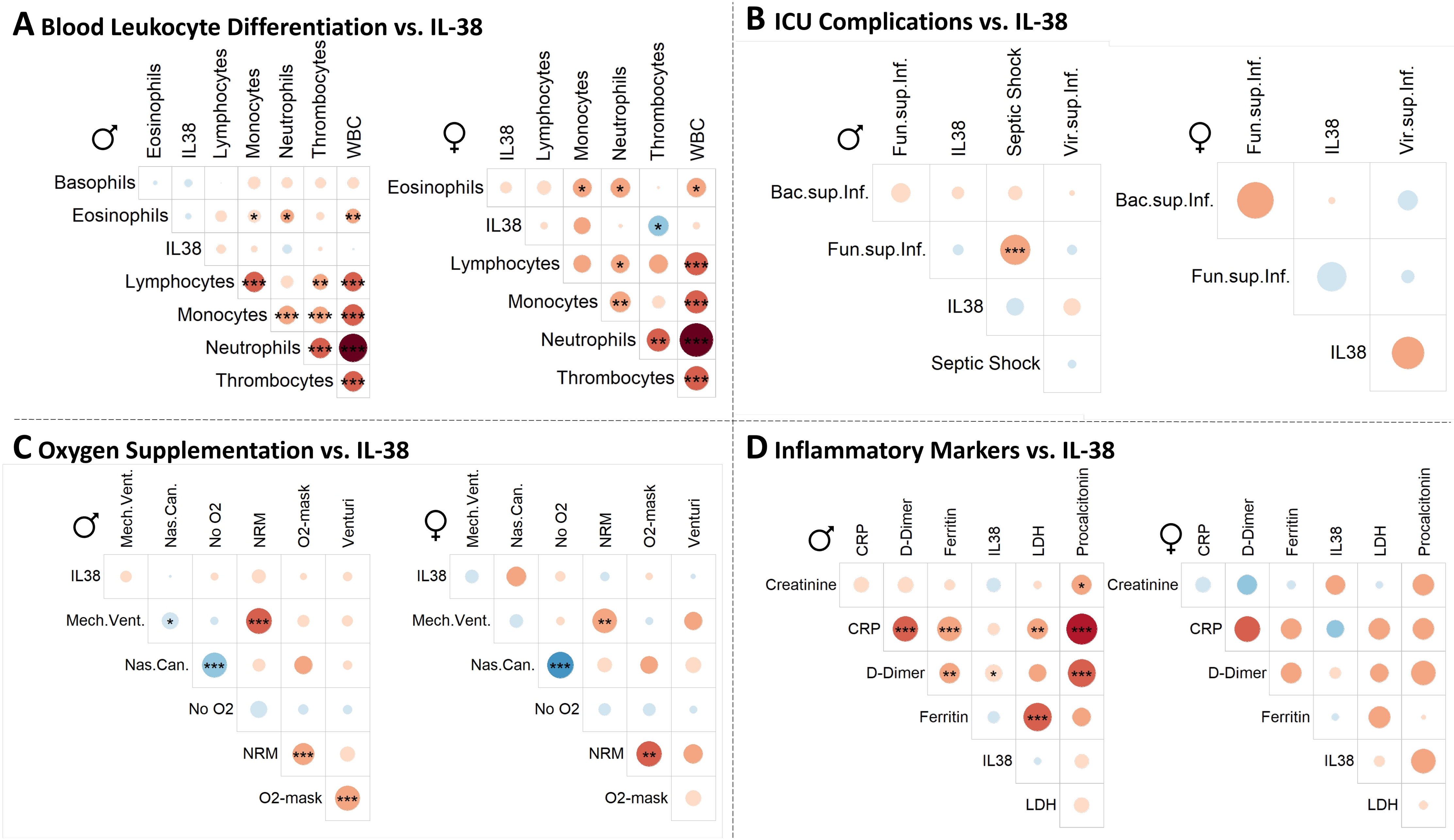
A Blood Leukocyte Differentiation vs. IL-38
♂
Basophils
Eosinophils
IL38
Lymphocytes
Monocytes
Neutrophils
Thrombocytes
WBC
♀
B ICU Complications vs. IL-38
Bac.sup.Inf.
Fun.sup.Inf.
Septic Shock
Vir.sup.Inf.
C Oxygen Supplementation vs. IL-38
Mech.Vent.
Nas.Can.
No O2
NRM
O2-mask
Venturi
D Inflammatory Markers vs. IL-38
Creatinine
CRP
D-Dimer
Ferritin
LDH
Procalcitonin
