# Supplementary material for: Exploratory analysis of interleukin‐38 in hospitalized COVID‐19 patients
Source: Immun Inflamm Dis. 2022 Oct 26;10(11):e712. doi: 10.1002/iid3.712 (PMC9601778; doi:10.1002/iid3.712)
Supplement: Supplementary file 4 — Supplementary information. [file IID3-10-0-s003.docx]

**Supplemental Information**

**Table S1.** Correlations between clinical parameters and hospitalisation period, ICU admission, and mortality.

Shown are Spearman correlation coefficients with FDR-adjusted and unadjusted p-values stratified by sex.

| Variable 1 | Variable 2  (n) | | Men |  | Variable 2  (n) | | Women |  |
| --- | --- | --- | --- | --- | --- | --- | --- | --- |
|  |  | Spearman r | Unadjust-ed *p*-value | Adjusted *p*-value |  | Spearman r | Unadjust-ed *p*-value | Adjusted *p*-value |
| Mortality | Creatinine  (125) | 0.25 | < 0.01 | 0.01 | Pro-Calci-tonin (31) | 0.48 | 0.01 | 0.04 |
| ICU Admission | CRP  (114) | 0.47 | < 0.01 | < 0.01 | CRP  (53) | 0.40 | < 0.01 | 0.03 |
|  | D-Dimer  (108) | 0.51 | < 0.01 | < 0.01 | D-Dimer  (48) | 0.56 | < 0.01 | < 0.01 |
|  | Ferritin  (113) | 0.33 | < 0.01 | < 0.01 | Ferritin  (53) | 0.33 | 0.02 | 0.09 |
|  | Pro-Calci-tonin (70) | 0.57 | < 0.01 | < 0.01 | Creatinine (53) | -0.28 | 0.04 | 0.11 |
|  |  |  |  |  | LDH  (50) | 0.30 | 0.04 | 0.11 |
| Hospitali-sation | D-Dimer  (108) | 0.32 | < 0.01 | < 0.01 | D-Dimer  (48) | 0.40 | 0.01 | 0.04 |
|  |  |  |  |  | Creatinine  (53) | -0.43 | < 0.01 | 0.02 |
|  |  |  |  |  | Ferritin  (53) | 0.29 | 0.04 | 0.11 |

**Figure S1.** Proximity extension assays of differential protein expression and principal component analysis between recovered and deceased COVID-19 patients.

On the right, volcano plots of three protein panels (Cardiovascular II (A), Inflammation (B and D), and Cardiometabolic (C)) are shown comparing differentially expressed proteins between deceased and recovered COVID-19 patients. Proteins are displayed as log2 fold change of expression plotted against FDR-adjusted p-values. Positive foldchanges indicate higher expression in deceased patients and negative foldchanges indicate lower expression in deceased patients. P-values < 0.05 are displayed in red. On the left, PCA plots of the same panels are shown. Deceased patients are depicted in red, recovered patients in blue. Panels A-C concern the validation cohort (Recovered_A_ n = 80, Deceased_A_ n = 15; Recovered_B_ n = 80, Deceased_B_ n = 15; Recovered_C_ n = 81, Deceased_C_ n = 16) and panel D the discovery cohort (Recovered n = 58, Deceased n = 26).

**Figure S2.** Correlation matrices of clinical parameters and IL-38.

Shown are correlation matrices of Spearman correlation coefficients and their significances for associations between blood leukocyte differentiation (A), ICU complications (B), oxygen supplementation (C), and inflammatory markers of disease (D) of the validation cohort stratified by sex. Men are on the left and women on the right in all panels, and IL-38 was an included in correlations of all clinical parameters. Correlations are highlighted in colour, red being a positive and blue a negative correlation. Colour intensity indicates the Spearman correlation coefficients (r). Correlations are highlighted in colour, red being a strong and blue a weak correlation and P-values are FDR-adjusted. Annotation: p < 0.05*, p < 0.01**, p < 0.001***. Women n = 56, Men n = 128. Abbreviations: WBC – whole blood count; Bac.sup.Inf. – bacterial super infection; Fun.sup.Inf. – fungal super infection; Mech.Vent – mechanical ventilation; Nas.Can – nasal cannula; NRM – non-rebreathing mask; CRP – C-reactive protein; LDH – lactate dehydrogenase.

**Figure S3.** Age and BMI distribution of discovery and validation cohort with corresponding healthy controls.

Shown are density plots of the age (A) and BMI (B) distribution of discovery (black) and validation (grey) cohort with corresponding sex- and age-matched control cohorts (yellow and white, respectively). Age is represented in years and body mass index (BMI).
